# Down regulation of the high-affinity IgE receptor associated with successful treatment of chronic idiopathic urticaria with omalizumab

**DOI:** 10.1186/1476-7961-9-2

**Published:** 2011-01-19

**Authors:** Michael C Saavedra, Sanjiv Sur

**Affiliations:** 1 Division of Allergy, Pulmonary, Immunology, Critical Care and Sleep, Department of Medicine, The University of Texas Medical Branch, 301 University Boulevard, Galveston, Texas, 77555, USA

## Abstract

Chronic idiopathic urticaria is a condition that is often controllable with antihistamine therapy. However, some patients have disease burden that is difficult to manage, non-responsive to antihistamines and often requires immunosuppressive medications such as corticosteroids or cyclosporine. We present here a study that demonstrates the effectiveness of omalizumab in treating this condition and the temporal relationship between improvement and down regulation of the high affinity IgE receptor (FcεRI). For this, blood samples were obtained from a symptomatic patient before each treatment and processed for flow cytometric analysis of FcεRI levels on the surface of blood basophils. Down regulation of FcεRI was observed in association with significant clinical improvement and discontinuation of immunosuppressive medications.

## Background

While approximately 20% of the population will experience an episode of acute urticaria at some point in their lifetime, only 0.1% will experience the scourge of chronic urticaria [1]. This disease is characterized by at least 6 weeks of almost daily episodes of intensely pruritic cutaneous wheals that typically last less than 24 hours and are not associated with residual pigmentation. Half of patients with chronic urticaria are thought to have this disease as a result of autoimmune phenomenon, while the remaining patients are designated as having "idiopathic" disease. It has been estimated that approximately 35-45% of patients possess autoimmune IgG antibodies that target the alpha subunit of FcεRI or, to a lesser extent, target directly the IgE antibody [2]. A link between thyroid autoimmunity and chronic urticaria has also been observed in a subset of patients [3]. Consequently, the evaluation of patients with chronic urticaria may include investigating for thyroid dysfunction and for the presence of microsomal antibodies and/or anti-thyroperoxidase antibodies.

Treatment of patients with chronic urticaria, autoimmune or idiopathic, involves targeting the H1 receptor with sufficient doses of antihistamines that will control the patient's symptoms. When symptoms can not be controlled with maximal doses of antihistamines, immunosuppressive medications such as corticosteroids or cyclosporine are often employed. However, the potential short and long term side effects from these medications make their use less than desirable for both the clinician and patient. Omalizumab is a recombinant monoclonal antibody that selectively binds to IgE and inhibits its binding to FcεRI on the surface of mast cells and basophils. The beneficial effects of this therapy in the treatment of moderate to severe persistent asthma have been well documented [4]. However, the off-label use of omalizumab for treatment of chronic urticaria has shown promise and represents an immunosuppressive sparing treatment option for patients with disease burden that is difficult to manage [5]. Omalizumab has also been shown in a previous study to significantly reduce symptoms in patients with documented chronic autoimmune urticaria [6]. Thus, omalizumab is increasingly becoming an accepted new treatment modality for use in patients with recalcitrant chronic urticaria.

## Case Presentation

A 51 year-old woman with a past medical history of well controlled asthma, allergic rhinitis and atopic dermatitis was referred to our university clinic complaining of chronic urticaria for the previous three years. She experienced almost daily episodes of hives that would last less than a day and were intensely pruritic. Prior to presentation in our clinic, she was treated by several physicians with various combinations of high dose first and second generation antihistamines without success. Montelukast offered no benefit when added to treatment with antihistamines. However, relief was obtainable with oral prednisone (20 mg/day) or cyclosporine (200 mg daily in divided doses). A biopsy was obtained which confirmed the diagnosis of true urticaria and ruled out urticarial vasculitis. During the course of her work up, a number of laboratory tests were ordered and were unrevealing as to the potential etiology of her hives (Table 1). She noted no association of symptoms with food or medications. Despite frequent monitoring, she was fearful of the potential toxic effects from cyclosporine after she experienced a transient decrease in renal function of unknown significance. Additionally, she was intolerant of prednisone when used at times in place of cyclosporine due weight gain and psychosis. In an effort to find a more tolerable and effective alternative, treatment was initiated with omalizumab 300 mg every two weeks. This dose was chosen based upon the severity of her symptoms and previous successful outcomes [5]. After the first treatment with omalizumab, the patient noted significant improvement. Over the course of the subsequent 2 weeks, she was able to wean cyclosporine down to 25 mg daily without experiencing an urticarial flare. This was the lowest tolerable dose required to prevent flares until the eighth treatment visit at which time she was able to completely withdraw from cyclosporine use. At the start of omalizumab treatment, she would experience only a generalized sensation of pruritus without visible lesions. This symptom was controlled initially with the addition of diphenhydramine 25 mg every 8 hours, and later with this medication used only on an as needed basis. Interestingly, a tolerance to the sedative effects of diphenhydramine developed after a few days of scheduled treatment as has been suggested by other authors [2]. During the course of the first 28 weeks of therapy she experienced only three episodes of hives that were easily managed. After this initial successful time period, her treatments were spaced to every three weeks with no further symptoms or complications. There were no immediate or late phase hypersensitivity reactions experienced by the patient during treatment with omalizumab.

**Table 1:** Laboratory values prior to treatment

Test Name	Result	Reference Range
IgE Level	191 IU/mL	0-180 IU/mL
Anti-FcεRI Ab	0.7%	0-5.0%
Thyroid Stimulating Hormone	1.23 mU/L	0.5-5.5 mU/L
Tryptase	5 ng/ml	2-10 ng/ml
Sedimentation rate	4 mm/hr	< 20 mm/hr
*Helicobacter Pylori *IgG Ab	Negative	

Ab, Antibody

**Figure 1:**
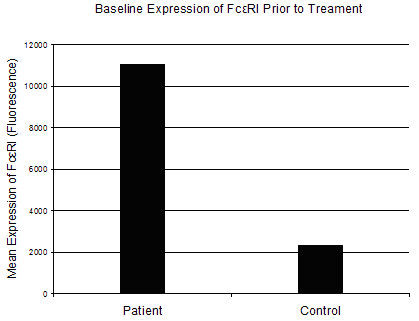
**Mean expression of FcεRI prior to treatment with omalizumab**. Peripheral blood was collected from the patient and a normal control subject prior to the patient's first treatment with omalizumab. Total FcεRI expression was examined in whole blood by flow cytometry using dual staining with basophil cell surface markers anti-CD123 (IL-3r) and anti-FcεRI.

**Figure 2:**
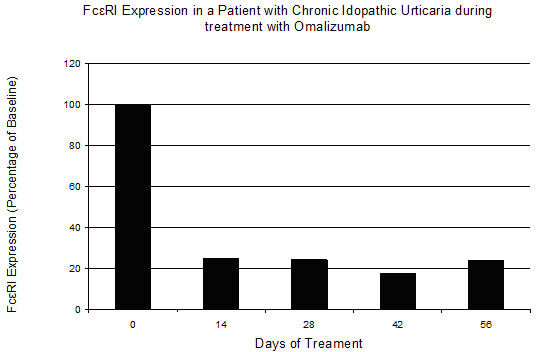
**Change in FcεRI expression during treatment with omalizumab**. Whole blood was collected from the patient prior to the first treatment with omalizumab (day 0) and prior to each subsequent treatment day. Total FcεRI expression was examined in whole blood by flow cytometry using dual staining with basophil cell surface markers anti-CD123 (IL-3r) and anti-FcεRI.

Prior to treatment with omalizumab and after consent was obtained, peripheral blood was obtained from the patient and from a control subject with no history of urticaria or allergic disease. After collection, samples were placed on ice, processed within three hours on the same day of collection and analyzed using dual staining flow cytometry to measure baseline expression of FcεRI on the surface of blood basophils (Figure 1). Additionally, expression of FcεRI was measured prior to each subsequent treatment over a 52 week period (Figure 2). For these experiments, FITC anti-FcεRI and PE anti-CD 123 antibodies (eBioscience, San Diego, CA), along with isotype controls, were added to whole blood. The sample was then treated with BD FACS Lysing Solution (BD Biosciences, San Jose, CA) to lyse the red blood cells. After a series of centrifugation and washing steps with staining buffer (1:10 dilution of PBS and 10% FBS), the cells were fixed with 2% paraformaldehyde and analyzed by flow cytometry.

When compared with the control subject, our patient displayed a five fold greater expression of FcεRI prior to treatment with omalizumab. After the first 14 days of treatment, there was an approximate 80% decrease in the expression of the high affinity IgE receptor that was maintained throughout the duration of treatment. This level of decrease is similar to previous published reports [7]. While mast cells represent the effector cell implicated in chronic urticaria, these experiments utilized antibodies for two surface markers found on the surface of basophils. It has been previously shown that treatment with omalizumab results in a reduction in free IgE and a decrease in FcεRI on blood basophils [8]. Previous studies have also reported that after treatment with omalizumab skin mast cells demonstrate a phenotypic shift and a reduction of surface FcεRI, albeit at a slower rate than is seen with blood basophils [9]. The patient in this study experienced significant improvement after the first treatment, though it was 14 weeks until she was able to completely withdraw from cyclosporine use altogether. This may be due to a slower response for achieving a decrease in mast cell numbers, mast cell function and/or mediator release. Indeed, regulation of mast cell survival is thought to be mediated in part by IgE-FcεRI dependent pathways [10]. While further studies are needed to fully understand the mechanism of efficacy with this new treatment modality, our study points to the importance of decreased FcεRI expression in this process.

## Conclusion

Treatment with omalizumab and the resultant down regulation of FcεRI expression is temporally associated with improvement of chronic idiopathic urticaria.

## Consent

Written informed consent was obtained from the patient for publication of this case report. A copy of the written consent is available for review by the Editor-in-Chief of this journal.

## Competing interests

The authors declare that they have no competing interests.

## Authors' contributions

MCS participated in the study design, carried out the sample collection, flow cytometry studies and drafted the manuscript. SS participated in the study design and coordination. All authors read and approved the final manuscript.
